# Fibromyalgia as a Disorder Related to Distress and its Therapeutic Implications

**DOI:** 10.1155/2012/950602

**Published:** 2012-03-05

**Authors:** Petra Schweinhardt, Mary-Ann Fitzcharles, Chad Boomershine, Charles Vierck, Muhammad B. Yunus

**Affiliations:** ^1^Alan Edwards Centre for Research on Pain, McGill University, Montreal, QC, Canada H3A 2B2; ^2^Boomershine Wellness Centers and Division of Rheumatology and Immunology, Vanderbilt University, Nashville, TN, USA; ^3^McKnight Brain Institute, University of Florida, Gainesville, FL 32611, USA; ^4^Section of Rheumatology, Department of Medicine, University of Illionois College of Medicine at Peoria, Peoria, IL 61656, USA

The concept of fibromyalgia has considerably evolved over the past two decades and now incorporates symptoms beyond pain, such as affective disturbances and fatigue [1]. This evolution of the clinical understanding of fibromyalgia, together with neurophysiological research showing abnormalities in central pain processing, has helped to establish the implication of the central nervous system (CNS) in fibromyalgia. The CNS, or more specifically the brain, is key when the organism is not (or no longer) capable to mount adaptive responses to stressors, that is, physical or emotional stimuli that threaten homeostasis. As a consequence stress turns into distress and homeostasis of the organism is no longer maintained. 

This special issue is centered on incorporating the concept of (dis-)stress into our understanding of fibromyalgia. A wide range of topics is covered, from clinical presentation to pathophysiology to treatment approaches, unified by the attempt to explore how stressors, together with individual vulnerability, impact on fibromyalgia. The paper by M. A. Fitzcharles and M. B. Yunus explains the clinical concept of fibromyalgia, demonstrating what fibromyalgia is from a clinical standpoint and what it is not. A call for standardization of assessment methods in the diagnosis of fibromyalgia and symptom severity as well as treatment outcomes is made by C. S. Boomershine, who provides a comprehensive evaluation of currently available tools. This paper might well serve as a starting point for discussions within groups like Outcome Measures in Rheumatology (OMERACT) with the goal of providing standardized assessment tools, which would clearly improve comparison of study results. The contribution by M. B. Yunus deals with the prevalence of fibromyalgia in other chronic pain conditions. On one hand, the overlap with other so-called functional pain syndromes is considered; on the other hand, the prevalence of fibromyalgia in diseases with clear (structural) organic cause is discussed. This approach is useful because it demonstrates that peripheral inflammatory and nociceptive events may trigger fibromyalgia symptoms in some patients while such events might be completely absent in patients presenting without any other organic disease. Then, Gracely et al. review commonalities and differences of fibromyalgia and depression. This is important for at least two reasons. First, depression is a frequent comorbidity of fibromyalgia. Second, fibromyalgia symptoms are regarded by some clinicians and/or researchers as a manifestation of a depressive disorder. The authors of this paper come to the interesting conclusion that although, in general, fibromyalgia may be more appropriately grouped with other functional pain disorders, certain subgroups of fibromyalgia patients with high level of psychological distress could be additionally or solely grouped with affective spectrum disorders. 

The paper by L. A. Low and P. Schweinhardt investigates how different stressors early in life, including pain itself as well as psychological trauma, contribute to increased vulnerability of stress and pain systems to further insult later in life. This is potentially important because it might explain pathophysiological changes observed in adult fibromyalgia patients. The following four contributions are concerned in detail with the pathophysiological alterations that might underlie the numerous symptoms of fibromyalgia. Ceko et al. carefully describe the experimental evidence for altered sensory processing that has been obtained in research settings. The authors discuss increased sensitivity to nociceptive stimulation, potentially altered endogenous pain modulation as well as increased sensitivity in response to other unpleasant sensory stimuli, which once more is indicative of a CNS disturbance rather than peripheral tissue abnormalities. Accordingly, they discuss neurobiological changes that might underlie fibromyalgia symptoms. S. Becker and P. Schweinhardt discuss in depth the possibility that dysfunctional neurotransmitter systems might be responsible for fibromyalgia symptoms. At first glance, this notion might seem at odds with the view that alterations in stress systems underlie fibromyalgia. However, the authors carefully explain how various symptoms of fibromyalgia could be explained by dysfunctional neurotransmitter systems and how alterations in the hypothalamic-pituitary-adrenal (HPA) stress system fit into this model. The paper by M. Martinez-Lavin is concerned with the important role of the body's second stress system in fibromyalgia. The paper illustrates how dysregulation of the sympathetic nervous system could be key in converting distress into pain. Light et al. review genetic polymorphisms that have been implicated in conveying susceptibility for fibromyalgia. In addition, they provide novel and existing data showing differential alterations of gene expression profiles in fibromyalgia and fibromyalgia comorbid with chronic fatigue syndrome. These results emphasize the importance of carefully subgrouping fibromyalgia patients. Furthermore, the genes identified include genes pertaining to the adrenergic system, to exercise-responsive metabolite sensing ion channels, and to sensory receptors possibly responding to muscle fatigue. Thereby they provide further evidence how autonomic dysregulation and vasoconstriction could be turned into ischemic muscle pain and fatigue. 

Treatment for fibromyalgia presently consists of a multimodal approach combining pharmacological and nonpharmacological interventions. Two papers in this special issue directly address the important topic of treatment, incorporating current information on pathophysiology in the choice of treatment. The first one by C. J. Vierck is built on the hypothesis that pain in fibromyalgia might be partly caused by insufficient perfusion of skeletal muscles, as mentioned in the preceding paragraph. The author focuses on physical exercise as a first-line treatment for fibromyalgia with the rationale that exercise can attenuate consequences of stress in the periphery, that is, vasoconstriction and resulting ischemic pain, as well as in the CNS. In the CNS, exercise could interrupt the reciprocal interactions between stress and systems that control autonomic regulation, mood, sleep, and possibly pain sensitivity. The paper by R. L. Woolfolk et al. in the special issue addresses a therapeutic intervention that directly targets the CNS. They report on using an individually administered form of cognitive behavioral therapy (CBT) with an emphasis on achieving competence in relaxation methods and improving patients' emotional self-awareness. The results of this form of CBT are impressive—almost two thirds of patients in the treatment group show 30% pain reduction compared to 5% in the control group that received treatment as usual. This is an encouraging start and justifies future studies focusing on determining which aspect of the individualized affective CBT drove those good treatment results by using more sophisticated control groups. 

The reader will appreciate some divergence between the different contributions to this special issue. For example, central sensitization has been classically regarded as a spinal process resulting from prolonged and/or high intensity afferent barrage related to peripheral injury. Then, some authors started to use the term relatively loosely by using it for any suspected CNS amplification of nociceptive processing. Recently, psychological stress has been shown to indeed modulate spinal glutamatergic neurotransmission, albeit possibly with different mechanisms than classical central sensitization [2]. These examples indicate that disturbance of homeostasis by different types of stressors, such as peripheral injury or psychological stress, might result in pain augmentation via different mechanisms. It remains to be determined whether there is a final common pathway of how different types of stressors that exceed the adaptive capacity of an individual lead to fibromyalgia. Although many questions regarding fibromyalgia are still unsolved, we have already come a long way. New diagnostic criteria have been developed and new etiological frameworks incorporating vulnerability to stress have been proposed. Research has greatly advanced our understanding of physiological alterations associated with fibromyalgia, including changes of the autonomic system and the CNS. Finally, nonpharmacological treatment approaches have been suggested based on the improved understanding of this challenging condition. Advancing our understanding of the mechanisms responsible for converting distress into pain and other symptoms of fibromyalgia further will likely have important implications for the development of new therapeutic perspectives.

Petra Schweinhardt

Mary-Ann Fitzcharles

Chad Boomershine

Charles Vierck

Muhammad B. Yunus
